# Recognition of Microbial Glycolipids by Natural Killer T Cells

**DOI:** 10.3389/fimmu.2015.00400

**Published:** 2015-08-04

**Authors:** Dirk M. Zajonc, Enrico Girardi

**Affiliations:** ^1^Division of Cell Biology, La Jolla Institute for Allergy and Immunology, La Jolla, CA, USA

**Keywords:** microbes, glycolipids, antigen-presentation, CD1d, TCR, NKT cells

## Abstract

T cells can recognize microbial antigens when presented by dedicated antigen-presenting molecules. While peptides are presented by classical members of the major histocompatibility complex (MHC) family (MHC I and II), lipids, glycolipids, and lipopeptides can be presented by the non-classical MHC member, CD1. The best studied subset of lipid-reactive T cells are type I natural killer T (iNKT) cells that recognize a variety of different antigens when presented by the non-classical MHCI homolog CD1d. iNKT cells have been shown to be important for the protection against various microbial pathogens, including *B. burgdorferi*, the causative agents of Lyme disease, and *S. pneumoniae*, which causes pneumococcal meningitis and community-acquired pneumonia. Both pathogens carry microbial glycolipids that can trigger the T cell antigen receptor (TCR), leading to iNKT cell activation. iNKT cells have an evolutionary conserved TCR alpha chain, yet retain the ability to recognize structurally diverse glycolipids. They do so using a conserved recognition mode, in which the TCR enforces a conserved binding orientation on CD1d. TCR binding is accompanied by structural changes within the TCR binding site of CD1d, as well as the glycolipid antigen itself. In addition to direct recognition of microbial antigens, iNKT cells can also be activated by a combination of cytokines (IL-12/IL-18) and TCR stimulation. Many microbes carry TLR antigens, and microbial infections can lead to TLR activation. The subsequent cytokine response in turn lower the threshold of TCR-mediated iNKT cell activation, especially when weak microbial or even self-antigens are presented during the cause of the infection. In summary, iNKT cells can be directly activated through TCR triggering of strong antigens, while cytokines produced by the innate immune response may be necessary for TCR triggering and iNKT cell activation in the presence of weak antigens. Here, we will review the molecular basis of iNKT cell recognition of glycolipids, with an emphasis on microbial glycolipids.

## Introduction

Microbial antigens can be recognized by various receptors of both the innate and adaptive immune system. While many receptors, especially innate immune receptors, bind their antigen in free form, T cell antigen receptors (TCRs), which are exclusively expressed on T cells, have evolved to recognize antigens when presented by dedicated antigen-presenting molecules (1). This feature distinguishes them from soluble immunoglobulins (Igs), which can bind to virtually any molecule in solution, even though, the Ig fragment that is responsible for antigen binding (Fab) is structurally very similar to the TCR. While exceptions to this rule exist, such as gamma delta (γδ) TCRs, which can bind antigens with or without antigen-presenting molecules (2), for the purpose of this review, we will exclusively focus on cells carrying the more well-characterized αβ TCRs.

T lymphocytes are key cells of the adaptive immune system. They recognize infection, initiate, and regulate immune responses, especially by controlling the activation of bystander immune cells (3). The hallmark of T cell activation is the direct binding of the TCR to an antigen that is presented by major histocompatibility complex (MHC)-encoded molecules. While classical MHC class I or II molecules are important for peptide presentation, non-classical MHC molecules, especially CD1, are required for glycolipid presentation to T cells (4–8). Other non-peptidic antigens, such as microbial vitamin B metabolites, can also be recognized by T cells when they are presented by the non-classical MHC I molecule, MR1 (9, 10).

## The CD1 Family

First identified in the late 1980s in the group of Cesar Milstein, CD1 is a group of MHC class I-like antigen-presenting molecules (11). CD1 proteins exhibit little or no polymorphism, in stark contrast to the MHC-encoded antigen-presenting molecules. However, the number of expressed CD1d genes varies widely by species. Humans express five functional isotypes (CD1a-e) (12), with CD1e being the only member that does not directly present antigens to T cells (13). Mice express only CD1d, while other species, such as guinea pigs, express multiple forms of the same isotpye, CD1b (14). CD1 molecules are conserved throughout vertebrate evolution (15) and have been identified to present lipids, lipopeptides, and glycolipids. While we will exclusively focus on the presentation and T cell recognition of glycolipids, it should be noted that the CD1-mediated presentation of phospholipids and lipopeptides is also well characterized (16–20).

## CD1 Structure

CD1 overall resembles MHC I molecules, where the heavy chain (MHC or CD1) non-covalently associates with β_2_-microglobulin (β_2_m) (4). The heavy chain can further be divided into three domains. The N-terminal α1 and α2 domains together form the antigen-binding site, while the α3-domain pairs with β2m to support the α1–2 platform. The CD1 binding groove is formed by two anti-parallel α-helices that sit atop an anti-parallel β-sheet platform. CD1 has evolved a hydrophobic antigen-binding groove, which is deeper than that of MHCI and well suited for the binding and presentation of hydrophobic molecules, such as lipids (4). For in-depth information about the structural details of different CD1 isoforms and species, see Ref. (21–32). Each CD1 protein has adopted isoform and species-specific binding pockets that differ in shape and size; however, all mammalian CD1 binding grooves contain the two major pockets, A′ and F′. While the A′ pocket is larger, donut shaped, and deeply buried, the F′ pocket is more open and accessible to the solvent. Each hydrophobic pocket generally binds one alkyl chain of a dual alkyl chain glycolipid, while the carbohydrate portion is located at the CD1d surface for TCR interaction. TCR recognition requires the proper presentation of the glycolipids by CD1d and TCR binding of the exposed carbohydrate epitope in conjunction with CD1d. The lipid backbone itself can also be in contact with the TCR to varying degrees; however, its main role is to anchor and orient the carbohydrate for T cell recognition (33). Previously, we assumed that the CD1d-binding groove is rather rigid and that each lipid will have to find the right fit inside both pockets. However, we now know that especially at the surfaces above both A′ and F′ pockets, subtle structural changes can be induced upon lipid and/or TCR binding (26, 33–35). Lipid-induced structural changes in mouse CD1d have only been observed by using synthetic glycolipids and whether natural lipids exist that have the same effect is currently unknown. However, lipid-induced structural changes especially around the F′ pocket greatly influence iNKT cell activation (33, 36) as this is the primary binding site for the TCR (37, 38).

## Lipid-Reactive T Cells

**Figure 1:**
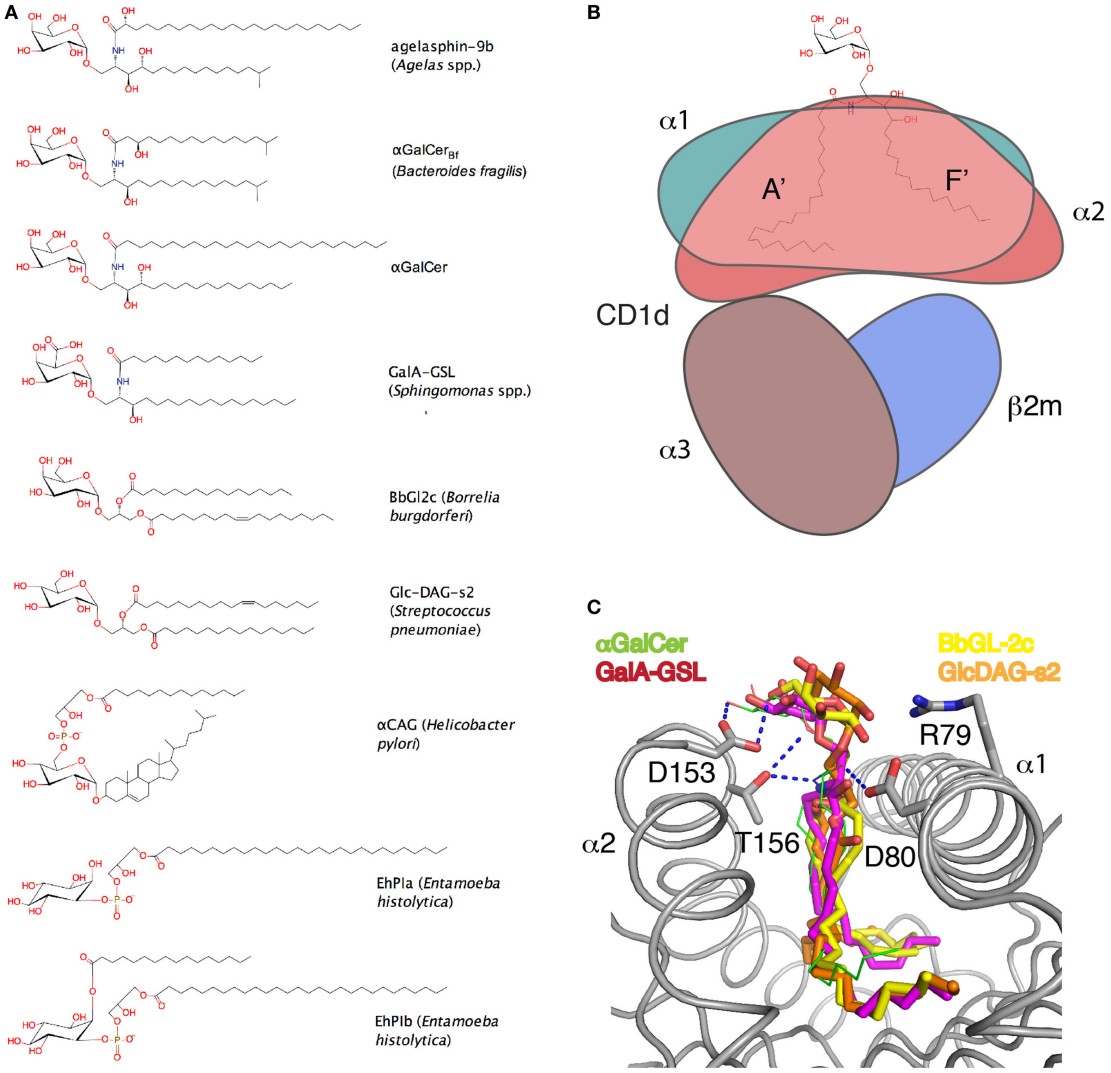
**Microbial glycolipids and their presentation by CD1d**. **(A)** Chemical drawings of microbial glycolipid ligands. **(B)** Cartoon of glycolipid presentation by CD1d. The α1–α2-domain (green, salmon shading) form the two major pockets A′ and F′ of CD1d that bind the lipid backbone, while the carbohydrate epitope is exposed. The α3-domain (brown) non-covalently binds β_2_-microblobulin (β_2_m, blue) and together supports the α1–α2-domain of CD1d. **(C)** Binding orientation of the different glycolipid headgroups superimposed in the CD1d binding groove. Hydrogen bonds between αGalCer and the CD1d molecule are shown as dashed lines.

Lipid-reactive T lymphocytes are a minor population compared to peptide reactive T cells but have been reported to influence the outcome of the immune response (3). Lipid-reactive T cell, especially those expressing an αβ TCR can be divided further based on their CD1 restriction and antigen-reactivity. Human group I CD1 (CD1a–c)-restricted T cells are generally considered diverse in their TCR repertoire and have often been reported to recognize mycobacterial antigens as well as self-antigens (39–46). Group II CD1 (CD1d)-restricted T cells, also called NKT cells, on the other hand, can further be classified into type I (Vα14*i*, iNKT) and type II NKT cells based on their TCR expression. Type I NKT cells are characterized by their evolutionarily conserved TCRα chain rearrangement [TRAV11–TRAJ18 (Vα14Jα18) in mice and TRAV10–TRAJ18 (Vα24Jα18) in human] and their reactivity toward the prototypical antigen α−galactosylceramide (αGalCer) (Figure 1), while type II NKT cells do not have a common antigen and represent all the remaining CD1d-reactive T cells that do not react to αGalCer (6, 47, 48). Recently, a population of CD1b-restricted T cells, called germline-encoded mycolyl lipid-reactive (GEM) T cells has been identified that similar to type I NKT cells, use a more restricted TCRαβ repertoire (predominantly TRAV1–2–TRAJ9) to bind mybobacterial antigens with high affinity (49). Type I NKT cells in particular have been identified as important in the protection against various microbial pathogens through direct recognition of microbial glycolipids.

## Microbial Activation of Type I NKT Cells

### Cytokine-mediated activation with and without TCR stimulus

**Figure 2:**
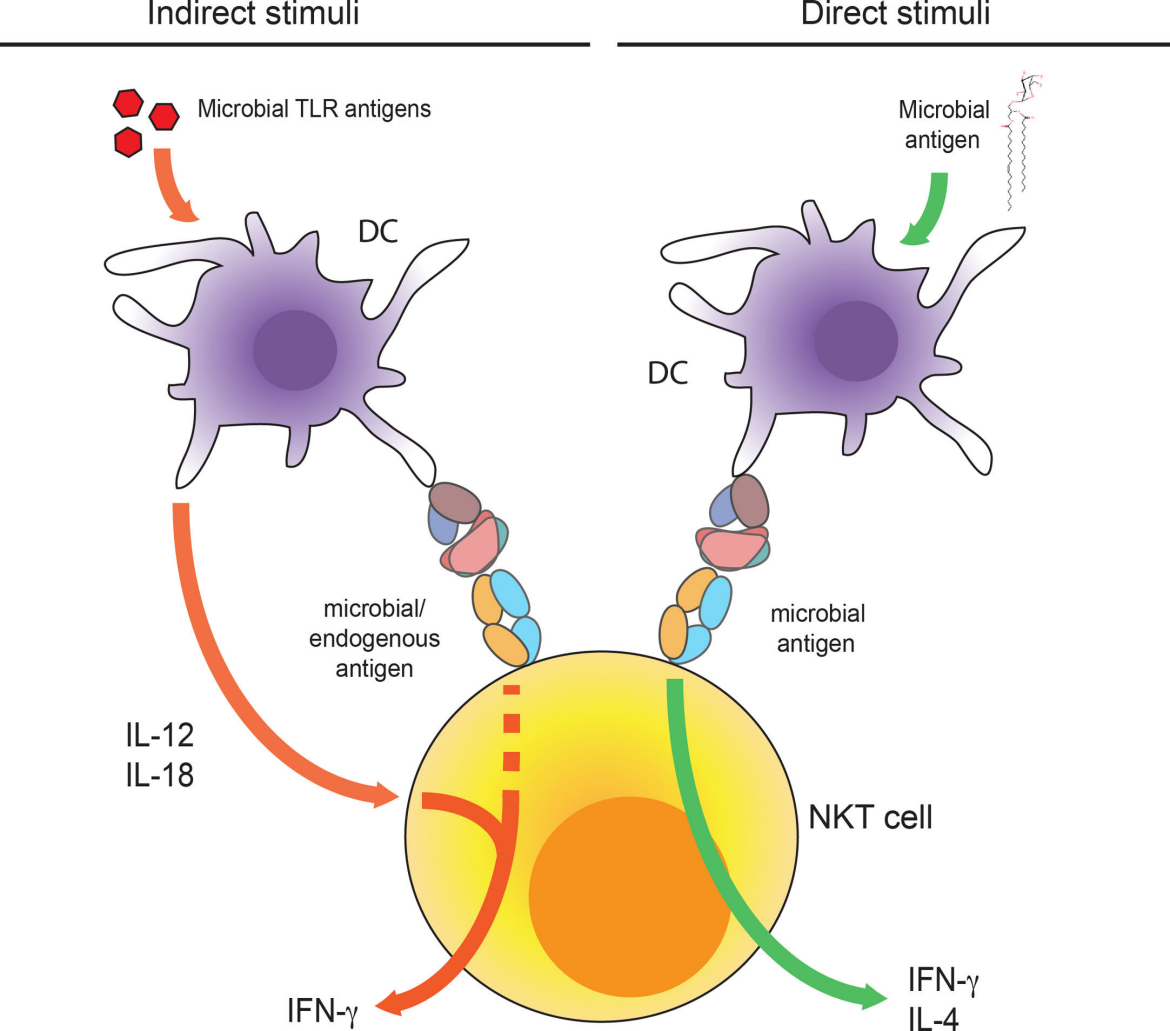
**Indirect and direct activation of NKT cells**. Dendritic cells produce IL-12 and IL-18 upon activation by TLR agonists that together with TCR engagement of weak microbial or self-antigens lead to the activation of iNKT cells (left pathway). DC presentation of microbial antigens can directly activate iNKT cells through TCR engagement (right pathway).

Type I NKT cells have been shown to participate in protection of mice from a variety of microbial parasites (3, 50, 51). While type I NKT cells expand upon infection, this is not necessarily due to the direct recognition of microbial antigens by the TCR (52). Furthermore, type I NKT cells can be activated *in vitro* and *in vivo* directly by cytokines, such as IL-12 plus IL-18 (53), or IL-12 alone, even in the apparent absence of a TCR signal (54) (Figure 2). Mouse cytomegalovirus infection leads to the activation of type I NKT cells in a CD1d-independent but IL-12-dependent manner, hinting to a protective role of type I NKT cells in viral infection (55, 56). Even in the case of bacterial infection, where foreign, microbial antigens are present, type I NKT cells can be activated with the help of cytokines, such as IL-12, and perhaps in some cases, in conjunction with the presentation of self-antigens rather than microbial antigens (57, 58). This has been demonstrated in the case of *Streptococcus pneumoniae* infection, where type I NKT cell activation is strongly dependent on IL-12, while CD1d deficiency greatly reduced but did not fully abrogate NKT cell activation (59).

### Glycolipid activation of type I NKT

The first antigen shown to activate type I NKT cells was α-galactosyl ceramide (αGalCer), which was isolated from a marine sponge in a screen for compounds that prevented tumor metastases in mice and changed by medicinal chemistry from the parental compound, Agelasphin-9b (Figure 1). αGalCer is now widely considered the prototypical antigen for human and mouse type I NKT cells. αGalCer is a glycosphingolipid, in which an α-anomeric galactose is connected to a ceramide backbone. The ceramide consists of a sphingoid base, which carries an N-amide-linked saturated C26 acyl chain. Interestingly, a new study identified α-glycosyl ceramides in immune cells in mice, where they could play an important role in the development of iNKT cells (60, 61).

αGalCer binds to CD1d with the C26 acyl chain in the A′ pocket and the sphingoid base in the F′ pocket (Figure 1). This binding orientation exposes the galactose moiety above the CD1d-binding groove for interaction with the TCR and subsequent NKT cell activation.

### Glycosphingolipids from *Sphingomonas* spp

The first identified and characterized microbial antigen for type I NKT cells was a glycosphingolipid from *Sphingomonas* bacteria. *Sphingomonas* are Gram-negative bacteria that lack lipopolysaccharide (LPS) and are highly abundant in the environment, including sea water (62, 63). Although *Sphingomonas* is not highly pathogenic, mice lacking type I NKT cells are defective for clearance of *Sphingomonas yanoikuyae* at early times after infection, while at later times, the bacteria was cleared without signs of any damage (64, 65). While the original TRAJ18^−/−^ mice used in those studies had a lower TCR repertoire, which could potentially contribute to some of the observed effects, a new mouse strain lacking iNKT cells is now available to assess the contribution of iNKT cells in host defense and other disease models (66, 67).

Similar to αGalCer, the antigen GalA-GSL also carried an α-linked sugar connected to a ceramide backbone (64, 68). However, instead of a galactose, the most potent antigen contained a galacturonic acid, while the ceramide lacked a hydroxyl group at C4 of the sphingoid base (Figure 1). In addition, instead of the C26 acyl chain found in αGalCer, GalA-GSL contains a much shorter C14 fatty acid.

### *Borrelia burgdorferi* galactosyl diacylglycerol antigens

*Borrelia burgdorferi* is a spirochete and the causative agent of Lyme disease. Mice lacking type I NKT cells were less capable of clearing *B. burgdorferi* and they were more subject to chronic joint inflammation (69–71). One week after bacterial infection, type I NKT cells were activated *in vivo* to produce cytokines, such as IFNγ and IL-4 (70). *B. burgdorferi* is the first example of a pathogenic microbe that contain glycolipid antigens that activates type I NKT cells, and it is also the first example showing that type I NKT cell antigens do not have to be glycosphingolipids (72). *B. burgdorferi* has abundant glycosylated diacylglycerols (73, 74) with an α-anomeric galactose sugar in the *sn-3* position of the glycerol. The *sn-1* and *sn-2* positions carry different acyl chains, most prominently palmitate (C16:0), stearate (C18:0), oleate (C18:1), and linoleate (C18:2) (Figure 1).

Using synthetic versions of the diacylglycerol antigen from *B. burgdorferi*, carrying different acyl chains at both *sn-1* and *sn-2* position, revealed the impact of the lipid backbone in type I NKT cell activation. The glycolipid, BbGL-2c (*sn-1*, oleate, *sn-2*, palmitate) proved to be stimulatory for mouse type I NKT cells, while BbGl-2f (*sn-1*, linoleate, *sn-2*, oleate) was the preferred antigen for human type I NKT cells (72, 75). The data suggested that despite having an α-anomeric galactose for TCR recognition, identical to αGalCer, the nature of the lipid backbone that anchors the glycolipid to CD1d can influence T cell activation. Although diacylglycerol lipids are less potent than sphingolipids, this finding is important since diacylglycerols are widely distributed in microbes.

### *Streptococcus pneumoniae* glucosyl diacylglycerol antigens

*S*.*pneumoniae* and Group B streptococcus are important pathogens responsible for pediatric and community-acquired pneumonia. α-glucosyl-containing diacylglycerol antigens (Glc-DAG)-s2, the main iNKT antigen found in these bacteria, was the first microbial antigen identified that did not carry a galactosyl moiety (76). Instead, it is composed of an α-linked glucosyl hexose linked to a diacylglycerol backbone. Interestingly, the *sn*-2 position of the glycerol carries an unusual vaccenic (C18:1, *cis*-11) acid, while the *sn*-1 position is occupied by palmitic acid (C16:0). The requirement for this unusual combination of sugar and fatty acid appears to be quite stringent, as the positional isomer carrying a vaccenic acid in position *sn-1* is not antigenic and that replacement of the glucosyl moiety with galactose did not restore antigenicity (36). Notably, Glc-DAG-s2 is also antigenic in human NKT cells (76), suggesting that the importance of similar synergies between lipid and polar portion of the streptococcal iNKT antigens is maintained in humans.

### *Bacteroides fragilis* glycosphingolipid antigens

*Bacteroides fragilis* is a commensal bacteria found in humans and mice, where it colonizes the gut, and is characterized by an unusually high percentage of sphingolipds in its membranes. iNKT antigens were reported in these bacteria, which structurally resemble the prototypical antigen αGalCer (Figure 1) (77, 78), while carrying features also found in the original antigenic sphingolipid isolated from marine sponges, such as Agelasphin-9b. These antigens were found to have either stimulatory or inhibitory effects on iNKT cells, playing a critical role in iNKT homestasis during development. While no structural information is currently available on how these antigens are presented by CD1d, their structural similarity to well-characterized sphingolipids suggests a conserved CD1d-binding mode, with the sphingosine chain bound in the F′ pocket and the fatty acid chain in the A′ pocket.

### *Helicobacter pylori* glucosyl cholesterol antigens

Glycolipid antigens derived from *Helicobacter pylori*, the bacterium associated with the etiology of gastritis and peptic ulcers, have been recently described as cholesteryl α-glucoside antigens (79). In particular, the lipid cholesteryl phosphatidyl α-glucoside (αCPG) was shown to bind to CD1d and be able to stimulate iNKT cells. Clinical severity of gastric atrophy upon infection of *H. pylori* was correlated with the expression of the microbial enzyme cholesteryl α-glucosyltransferase (αCgT), which generates the iNKT cell antigen cholesteryl α-glucoside (79). Jα18-deficient mice lacking iNKT cells have an elevated burden of *H. pylori*, suggesting a protective role of iNKT cells against certain clinical isolates of *H. pylori*. While this study did not address whether the phosphatidyl moiety or the cholesterol moiety binds inside the CD1d-binding groove, unpublished data form our own lab indicated that the mouse CD1d can in fact bind cholesterol derivatives. How such a bulky and rigid lipid moiety is bound within the CD1d-binding groove is currently unclear and will require further structural characterization.

### *Entamoeba histolytica* acylated lysophosphatidyl inositol antigen

Another microbe that contains unusual iNKT cell antigens is *Entamoeba histolytica*, an intestinal protozoan parasite that causes amebiasis, resulting in significant morbidity and mortality worldwide (80). Clinical manifestations include liver abscesses, and animal models of experimental amebic liver abscess (ALA) demonstrated a role for IFN-γ in the control of *E. histolytica* invasion. As iNKT cells are predominantly located in the liver of mice and potently produce copious amounts of IFN-γ, it is not surprising that Jα18^−/−^ mice have considerably larger liver abscesses compared to wildtype mice, indicating a protective role of iNKT cells in parasite control. The iNKT cell antigen was generated upon lysosomal processing of a lipopeptidophosphoglycan to the active form EhPIb, 1-*O*-[(28:0)-lyso-glycero-3-phosphatidyl-]2-*O*-(16:0)-inositol (81). This lipid correlates with a model in which the C28:0 chain binds in the A′ pocket of CD1d, while the inositol headgroup is exposed above the CD1d-binding groove and the inositol-linked C16 fatty acid inserts into the F′ pocket of CD1d. However, no published data using a synthetic version of this antigen are available to date, which would be important to address the question of how such a structure binds to CD1d to activate iNKT cells.

## Antigen Loading and Processing

Lipid antigens are generally water-insoluble and if not bound to proteins themselves, will be embedded into cellular membranes. As a consequence, there has to be an active process in which lipids are extracted from the membranes of APCs and/or transferred from another protein into the binding groove of CD1d. While some antigens, such as αGalCer, can be loaded directly into CD1d molecules on the cell surface, antigen presentation generally is enhanced by the internalization of glycolipids into acidic endosomal compartments (82). As cell-surface expressed CD1 recycles back through cellular compartments, CD1 encounters lysosomal lipid transfer proteins, such as saposins A–D (83–87), which facilitate the transfer of glycolipids into CD1. Saposins can extract lipids from membranes, directly bind them in hydrophobic pockets, and transfer them onto CD1d. However, the mechanism of lipid transfer and whether saposins directly bind CD1d has not been well studied. In addition to saposins, a variety of cells, including DCs, secrete apolipoprotein E, which enhances the presentation of glycolipid antigens to iNKT cells because it can interact with glycosphingolipid antigens and enhance their uptake (88). Furthermore, microsomal triglyceride transfer protein (MTTP), an ER chaperone protein, has been reported to assist in lipid loading into nascent CD1d, as it passes through the secretory pathway on the way to the cell surface (89). As a result, endogenous lipids are found associated with cell-surface expressed CD1d.

In addition to lipid loading, lipid processing can also occur in acidic compartments. Using the synthetic glycosphingolipid antigens Gal(α1,2)αGalCer, carbohydrate antigen processing was demonstrated in APCs (90). Gal(α1,2)αGalCer itself is unable to directly activate iNKT cells but once the terminal galactose is removed by the lysosomal enzyme α-galactosidase A, thereby generating the highly antigenic monosaccharide, αGalCer, iNKT cell activation could be observed (90). Processing of microbial antigens has also been observed for the *Entamoeba histolytica* antigen, EhPIb. However, in contrast to the removal of a carbohydrate, removal of a fatty acid through a phospholipase (PL) is necessary to generate the antigen. Another example of microbial antigen processing is provided by hexamannosylated phosphatidyl-myo-inositols (PIM_6_), from *Mycobacterium tuberculosis*, which is presented by CD1b. PIM_6_ is processed to the dimannoside form PIM_2_ by α-mannosidase, a process that is greatly enhanced by CD1e (13). Also, recognition of iGb3 is believed to require processing from iGb4 by an enzyme or enzymes having the β-subunit found in lysosomal hexosaminidases A and B (91).

In summary, processing of antigens can occur both in the carbohydrate moiety, as well as the lipid backbone and appears to constitute a common feature of both endogenous and microbial antigens.

## Structural Basis of Glycolipid Presentation by CD1d

While the list of NKT cell antigens is rapidly expanding, most structural work on the presentation of glycolipids by CD1d has focused on dual chain lipids, such as diacylglycerol lipids or sphingolipids. Only limited structural data are available on the presentation of lysolipids, which are characterized by possessing only one alkyl chain, or other types of lipids, such as cholesterol derivatives. The few current examples of lysolipids include the presentation of lyso-PC by human CD1d, which is an antigen for iNKT cells and lyso-sulfatide by mouse CD1d, which is an antigen for a subset of type II NKT cells (92, 93). Both single chain lipids appear to bind in the F′ pocket of CD1d but activate distinct NKT cell populations. Lysophospholipids are derived from diacylglycerol lipids upon PL A1, A2, or B digestion, generating lysolipids with a single fatty acid located either at the *sn*-1 (PLA2, B) or *sn*-2 (PLA1, B) position. However, the current list of microbial lysolipids able to activate iNKT cells is limited. While *Entamoeba histolytica* contains a lysolipid with a fatty acid at the *sn*-1 (EhPIa), only the lipid that contains an additional fatty acid at the inositol moiety (EhPIb) appears to be antigenic for iNKT cells (Figure 1) (81).

**Figure 3:**
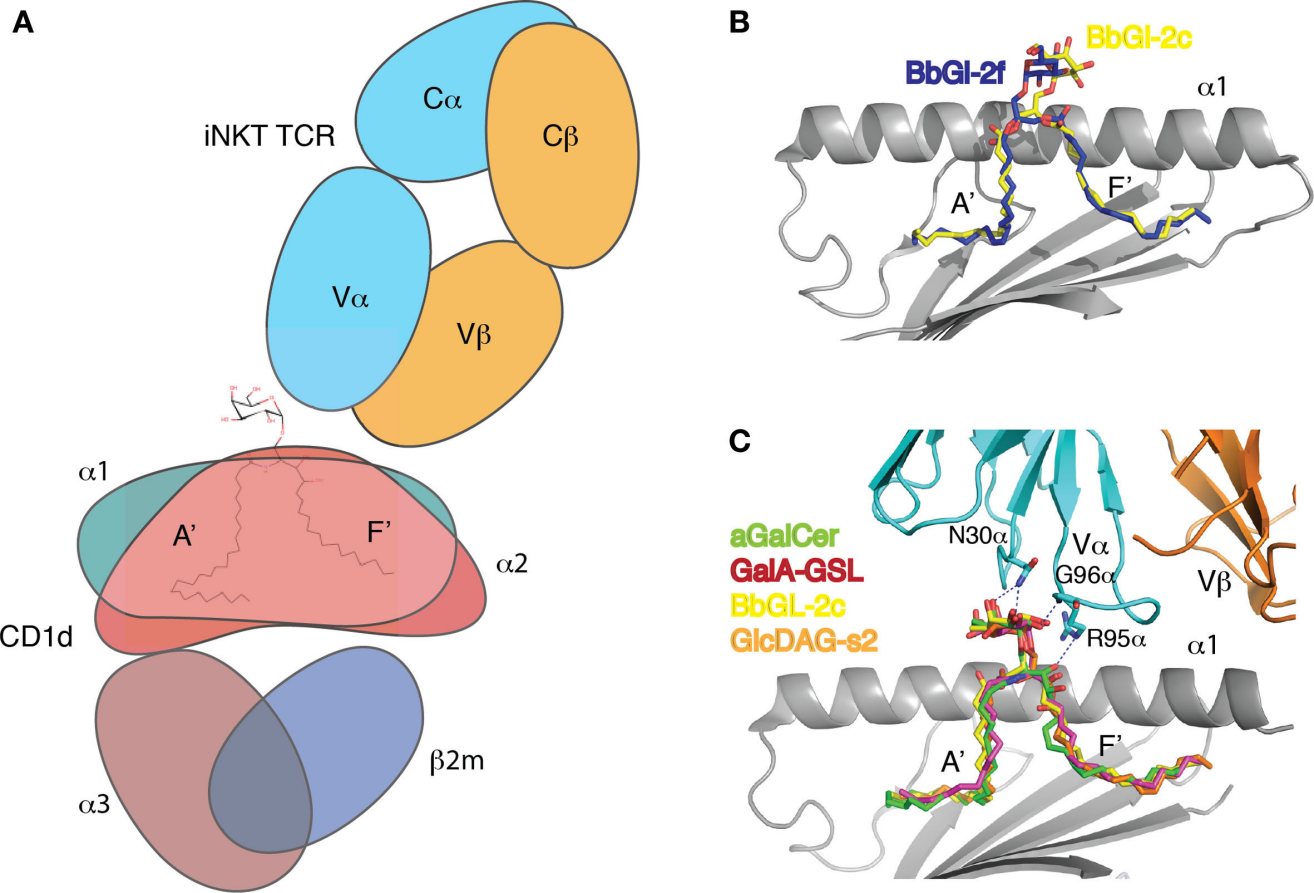
**TCR recognition of CD1d-presented glycolipids**. **(A)** Cartoon representation of the CD1d–αGalCer–Vα14Vβ8.2 TCR ternary complex. **(B)** Individual fatty acids can affect the binding orientation of diacylglycerolipid antigens. BbGl-2c binds with the *sn-1*-linked oleic acid in the A′ pocket, while BbGL-2f inserts its *sn-2* linked oleic acid. This affects presentation of the galacotse moiety. **(C)** The glycolipids are exclusively contacted by CRD1α and CRD3α residues of the Vα14 chain of the TCRα, while the Vβ8.2 chain is offset to the C-terminal end of the α1-helix, and only contacts CD1d directly. Note that all glycolipids superimpose well as they are molded into the same position by the TCR.

Microbial dual alkyl chain lipids binding to CD1d have been well characterized, both for ceramide-based glycosphingolipids and for diacylglycerol-containing glycolipids (33, 36, 64, 68, 72, 75, 76, 94, 95). We now know that sphingolipids bind in a conserved orientation to CD1d, with the longer acyl chain (typically up to C26) filling the larger A′ pocket, and the shorter sphingoid base (~C18) filling the smaller F′ pocket (96, 97). This binding orientation is maintained even when the fatty acid or the sphingosine moiety is truncated to eight or nine carbons, respectively, which would allow for a reversed binding orientation (26, 98). The binding orientation is likely orchestrated by a network of conserved H-bond interactions between the core mouse CD1d residues, Asp80, Asp153, and Thr156 (Asp151 and Thr154 are the equivalent residues in human CD1d) and the polar moieties of the rigid ceramide backbone (Figure 1C). If the fatty acid chain is too short to fill the A′ pocket completely, spacer lipids, such as a C16:0 fatty acids (palmitic acid), are recruited to occupy the remainder of the pocket, at least for proteins that were recombinantly expressed in insect cells (26, 95, 99). Spacer lipids have also been observed in the F′ pocket for glycosphingolipids, such as OCH, where the sphingoid base had been shortened (35, 98). The conserved binding orientation places the carbohydrate moiety in a rather similar position for TCR engagement. The first identified microbial antigens for iNKT cells were from Sphingomonas spp., which contained the glycosphingolipids α-glycuronosylceramide (containing either a glucuronic or galacturonic moiety). Structural data revealed that the overall binding to CD1d was similar compared to αGalCer, with slight different interactions based on the lack of the 4-OH group of the sphingoid base. This resulted in a slightly deeper binding of the sphingoid base inside the F′ pocket and a concomitant loss of well conserved electron density for the galacturonic acid headgroup at the CD1d surface (95). This less well-defined presentation of the headgroup was likely the major determinant for the reduced antigenicity compared to αGalCer and a reduced TCR-binding affinity (33). Interestingly, a subset of non-canonical Vα10 NKT cells had been described earlier that can also recognize αGalCer but have a preference for glucose-containing antigens, such as αGlcCer, and the microbial antigen, α-glucuronosylceramide. Despite sharing only 40% sequence conservation with the Vα14 chain, the Vα10 NKT cells have a CD1d-docking mode similar to type I NKT cells (100). iNKT cell antigens that contain ceramide backbones are generally more potent than antigens that are based on a diacylglycerol backbone. In fact, the fine structure of the diacylglycerol backbone greatly influences the potency of iNKT cell agonists. While the carbohydrate moiety is connected to the *sn*-3 position of the glycerol, the two fatty acids occupy the *sn*-1 and *sn*-2 position. This leads to a greater diversity in the lipid backbone of diacylglycerol lipids, as the fatty acids can vary in length and saturation. During the structural characterization of *B. burgdorferi* α-galactosyl diacylglycerolipid binding to mouse CD1d, we observed two binding orientations of the diacylglycerolipid backbone. The two antigens, BbGl-2c and BbGl-2f, bind with the oleic acid (C18:1) in the A′ pocket and the palmitic acid (C16:0, BbGl-2c) or linoleic acid (C18:2, BbGl-2f) in the F′ pocket. However, as the oleic acid is at the sn-1 position in BbGL-2c but in the sn-2 position on BbGL-2f, this results in the reversed binding orientation (Figure 3B). It has been demonstrated that only BbGL-2c can potently activate mouse iNKT cells, suggesting that the reversed binding orientation observed for BbGL-2f renders the antigen non-antigenic, even though the same galactose epitope is presented (75). However, presentation of this galactose moiety above the CD1d-binding groove is altered, suggesting that the fine positioning of the carbohydrate moiety at the TCR interface by the precise structure of the lipid backbone greatly affects antigenicity. It is intriguing to speculate that microbes can synthesize specific glycolipids in an attempt to invade immune recognition by iNKT cells; however, both BbGl-2c and BbGl-2f are abundantly expressed by *B. burgdorferi* (73).

*Streptococcus pneumoniae* contains α-Glc-DAG, in contrast to the galactose or galacturonic acid moieties found in *B. burgdorferi* and *Sphingomonas* spp. Glucose-containing glycolipids are generally considered as weaker antigens based on early studies using αGalCer analogs (101). The precise lipid structure contained an *sn*-3-linked α-glucose, a palmitic acid at *sn*-1, and a cis-vaccenic acid (C18:1, *cis*-11) at *sn*-2 (Glc-DAG-s2) (76). Replacing the glucose with a galactose surprisingly resulted in a glycolipid that was not able to activate murine iNKT cells (Gal-DAG-s2) (36). Similar observations were made when the cis-vaccenic fatty acid was replaced against oleic acid (C18:1, *cis*-9), which effectively only shifts the unsaturation two carbons toward the end of the chain, or when the cis-vaccenic acid was linked to the *sn*-1, rather than *sn*-2 position of the glycerol (36, 76). As such, the streptococcal antigen exhibited a unique interplay between the lipid backbone, the precise location of the unsaturation at the s*n*-2 linked fatty acid, and the glucose moiety. Presentation of this glycolipid by CD1d shared similarities with the presentation of the borrelial antigen BbGL-2c, which had the oleic acid at the *sn*-1 position. While both lipids bound in opposite orientations inside the CD1d-binding pocket, both the galactose and the glucose moiety were presented in a more upright tilted orientation to the TCR. This was a direct consequence of loss of intimate H-bond interactions with the core residue Asp153, which binds the 2″- and 3″-OH of αGalCer (Figure 1C).

## T Cell Recognition of CD1d–Glycolipid Complexes

In recent years, structural studies shed light on the recognition of the different microbial glycolipids by the Vα14Vβ8.2 TCR of murine iNKT cells (33, 36). Similar to the structure of the Vα14Vβ8.2 TCR bound to mCD1d-αGalCer (38), the microbial antigens are contacted by the TCR using a highly conserved binding chemistry. The TCR binds with the invariant Vα14Jα18 chain directly above the carbohydrate headgroup with a footprint centered above the F′ pocket. In stark contrast to TCR recognition of pMHC, the TCR beta chain does not participate in direct antigen recognition. The highly variable complementarity-determining region (CDR)3β loop exclusively contacts CD1d and is involved in controlling autoreactivity (102). The antigen is recognized by the TCR alpha chain, specifically CDR1α and CDR3α. In addition, certain antigens, such as isoglobotrihexosyl ceramide (iGb3) or phosphatidyl inositol (PI), are also contacted by the framework residue, Lys68, while αGalCer analogs with aromatic groups attached to the 6″-OH of the galactose can be in contact with CDR2α residue Gln52 (103–105). CDR1α exclusively contacts the headgroup, while CDR3α contacts both headgroup, the hydroxyls of the lipid backbone, and the F′ roof, indicating that the overall recognition and binding orientation is dominated by CDR3α (Figure 3). For αGalCer, the 3″ and 4″-OH groups of the galactose epitope make a hydrogen bond to Asn30 of CDR1α, while the 2″-OH interacts with the backbone nitrogen of Gly96 of CDR3α. Arg95 contacts the 4′-OH of the sphingoid base. All or most of those interactions are also observed in the microbial glycolipid structures but depending on the type of carbohydrate, the interaction with the axial 4″-OH can be lacking (e.g., in glucose, the 4″-OH is in equatorial configuration, pointing away from the TCR). Also, depending on the nature of the lipid backbone, the interaction between Arg95 of CRD3α and 4′-OH of the sphingoid base is not always formed. Surprisingly, while the carbohydrate moieties of three two microbial lipids, GalA-GSL, BbGL-2c, and Glc-DAG-s2, are presented by CD1d in different orientations before TCR engagement, all the lipid headgroups superimpose well with that of αGalCer after TCR binding. The combined biophysical and structural data revealed the basis of why αGalCer is such a potent antigen and high affinity ligand for the TCR. The TCR binds αGalCer using a lock and key mechanism, while microbial antigens, especially the carbohydrate headgroups have to be re-oriented by the TCR to allow for the conserved binding footprint on CD1d (33, 37, 106, 107). One could argue that the lipid structures of the analyzed microbial antigens are similar to αGalCer, despite the obvious differences in the lipid backbones. As such, the TCR not surprisingly binds to those antigens using a conserved binding chemistry. However, the conserved binding chemistry of the TCR has also been observed in the more complex β-anomeric glycolipid, iGb3, where the TCR completely flattens the trihexosyl group to form similar interactions with the β-anomeric glucose. In fact, the β-anomeric glucose is molded upon TCR binding into a position where it mimics the observed flat binding of α-anomeric carbohydrates (104, 108). TCR-binding kinetics also correlates well with the structural change that the TCR induces in the ligands upon binding to the CD1d–glycolipid complexes. All the microbial antigens that are not based on a ceramide backbone and as such differently presented by CD1d have a 10× or more reduced TCR association rate. Surprisingly, however, the TCR dissociates 70× faster from GalA-GSL compared to αGalCer. While both antigens are presented in a similar orientation, and also form the same number of H-bond interactions, we wondered whether there are any structural changes in CD1d. Indeed, as previously reported, the short chain αGalCer analog PBS-25 induces the closure of the F′ roof upon binding to CD1d, while the F′ roof is not closed by any of the microbial antigens (26, 33, 36). The F′ roof is the major binding site for the TCRα chain and upon binding of the TCR, the F′ roof is formed regardless of the bound glycolipid. That correlates with a model in which the TCR induces a structural change in CD1d for the TCRα chain to bind to, and the necessary binding energy to keep the roof closed is taken out of the TCR binding energy. As a result, the TCR dissociates faster from CD1d–glycolipid complexes that do not have a preformed F′ roof (33, 36). Further mutational studies targeting individual residues within the F′ roof highlighted the importance of this roof in the overall stability of the ternary complexes, without affecting TCR association rates, suggesting a two step-binding mechanism in which the antigen is bound first and then the CD1d molecule (36).

## Conclusion

The TCR of iNKT cells can recognize a vast range of antigens. While novel microbial antigens for iNKT cells continue to be identified, a clear structural pattern has emerged that the TCR recognizes with preferred specificity. The pattern consists of an α-anomeric monohexosyl sugar, linked to a lipid backbone that can either be based on a ceramide, a diacylglycerol, or potentially a cholesterol moiety. The type of sugar that is preferentially recognized would depend on the precise structure of the lipid backbone but generally contains a galactose, glucose, or derivatives thereof. While it is still difficult to predict which DAG backbone would give rise to iNKT cell antigens, the binding of glycosphingolipids is more conserved and many interactions with the TCR can now be modeled with confidence. Deviation from this pattern can be compensated by the unique binding properties of the TCR, with regards to the structural changes that it can induce in both lipid headgroup orientation and in CD1d. Those changes would be reflected in a lower TCR binding affinity and potency. As such, the most potent glycolipid antigens for iNKT cells, regardless of source, are glycosphingolipids followed by α-glycosyl diacylglycerolipids. Binding of other types of lipid antigens has not been structurally assessed but will be the focus of continuing studies, since many microbes also produce glycolipids that deviate from the known structures.

## Conflict of Interest Statement

The authors declare that the research was conducted in the absence of any commercial or financial relationships that could be construed as a potential conflict of interest.
